# Salivary miR-30c-5p as Potential Biomarker for Detection of Oral Squamous Cell Carcinoma

**DOI:** 10.3390/biomedicines9091079

**Published:** 2021-08-24

**Authors:** Nikolay Mehterov, Boyan Vladimirov, Andrea Sacconi, Claudio Pulito, Marcin Rucinski, Giovanni Blandino, Victoria Sarafian

**Affiliations:** 1 Department of Medical Biology, Medical University-Plovdiv, 4002 Plovdiv, Bulgaria; victoria.sarafian@mu-plovdiv.bg; 2 Research Institute, Medical University-Plovdiv, 4002 Plovdiv, Bulgaria; 3 Department of Maxillofacial Surgery, Medical University-Plovdiv, 4002 Plovdiv, Bulgaria; boyan.vladimirov@mu-plovdiv.bg; 4 UOSD Clinical Trial Center, Biostatistics and Bioinformatics, IRCCS Regina Elena National Cancer Institute, 00144 Rome, Italy; andrea.sacconi@ifo.gov.it; 5 Oncogenomic and Epigenetic Unit, IRCCS Regina Elena National Cancer Institute, 00144 Rome, Italy; claudio.pulito@ifo.gov.it (C.P.); giovanni.blandino@ifo.gov.it (G.B.); 6 Department of Histology and Embryology, Poznan University of Medical Sciences, 61-781 Poznan, Poland; marcinruc@ump.edu.pl

**Keywords:** miRNA, saliva, OSCC, biomarkers

## Abstract

The levels of different classes of extracellular RNAs (exRNAs) remain stable in bodily fluids. The detection of either enriched or depleted specific subsets of salivary microRNAs (miRNAs) has the potential to serve as a non-invasive approach for biomarker development. Thus, salivary miRNAs have emerged as a promising molecular tool for early diagnosis and screening of oral squamous cell carcinoma (OSCC). Total RNA was extracted from saliva supernatant of 33 OSCC patients and 12 controls (discovery set), and the differential expression of 8 cancer-related miRNAs was detected by TaqMan assay. Among the screened miRNAs, miR-30c-5p (*p* < 0.04) was significantly decreased in OSCC saliva. The same transcriptional behavior of miR30c-5p was observed in an additional validation set. miR-30c-5p showed a significant statistical difference between cases and controls with areas under the curve (AUC) of 0.82 (95% CI: 0.71–0.89). The sensitivity and the specificity of miR-30c-5p were 86% and 74%, respectively. The target identification analysis revealed enrichment of miR-30c-5p targets in p53 and Wnt signaling pathways in OSCC. Additionally, the miR-30c-5p targets had clinical significance related to overall survival. In conclusion, these findings show that downregulated miR-30c-5p has the potential to serve as a novel, non-invasive biomarker for early OSCC detection.

## 1. Introduction

Oral cancer is the most frequent malignant neoplasm of the oral cavity. Around 90% of all oral malignancies histologically originate from squamous epithelium and are subsequently classified as oral squamous cell carcinoma (OSCC). OSCC is unfortunately still associated with a poor survival rate [1]. When the disease is detected at an early stage (T1), the 5-year survival rate can reach up to 80%. In contrast, diagnosis at later stages (T3 or T4) dramatically decreases the chances for survival to 20–40% [2,3,4,5]. Therefore, there is an urgent need to find a quick, affordable and non-invasive approach that would help to detect and diagnose OSCC at an early stage, before metastatic spread.

Clinical oral examination followed by biopsies of suspicious lesions and histopathological evaluation still remains the golden standard for diagnosing OSCC. However, this procedure is invasive, causes discomfort, and requires time for the processing and examination of the tissues [6,7,8]. Another disadvantage lies in the fact that accessibility to deeply localized recurrences is quite challenging and these recurrences might require repetitive biopsies to monitor genetical changes that might occur during disease progression. Therefore, it is essential to develop a non-invasive and reliable, cost-effective diagnostic method that can accurately identify and distinguish OSCC at its early stages or when it reoccurs.

Salivary diagnostics has attracted particular attention as saliva is a biological fluid in direct contact with oral cancer tissues. Saliva can be easily collected in a non-invasive way and offers certain advantages compared to repetitive blood sampling, as the latter is physically invasive and could add excessive stress and pain to patients. This fluid contains desquamated epithelial cells as well as a broad range of secreted biomolecules such as cell-free DNA and RNA (mRNA, miRNA, lncRNA), proteins (cytokines), hormones, and several metabolites [9,10,11,12]. All of these biomolecules appear to enter the saliva through various sources, including the three major and hundreds of minor salivary glands, gingival crevice fluid, and desquamated oral epithelial cells.

In recent years, published data underlining the role of miRNAs in initiation and progression of different malignant diseases have increased exponentially [13,14,15]. Data obtained by different sequencing studies suggest that the number of miRNAs encoded by the human genome is above one thousand [16]. These small (19–24 nucleotides) non-protein coding RNA molecules regulate the gene expression in a post-transcriptional manner by translational inhibition or mRNA degradation [17]. Moreover, their stability and tissue- and disease-specific expression pattern in human peripheral blood and bodily fluids provide miRNAs with ideal biomarker properties for early diagnosis and prognosis of cancer [18]. Recent studies have suggested that thousands of miRNAs are present in salivary exosomes and they can be used as minimally invasive diagnostic biomarkers to detect OSCC and other malignancies [19,20,21,22,23,24].

In the present study, the transcription levels of a panel of 8 cancer-related miRNAs in the saliva of both OSCC patients and healthy controls were assessed. Among them miR-30c-5p and miR-34-3p were found to be underexpressed in oral cancer saliva. Further validation in a larger patient cohort confirmed that one of them, miR-30c-5p, showed the same transcriptional behavior. ROC curve analysis also demonstrated that miR30c-5p holds significant potential to differentiate between OSCC cases and controls. The target identification analysis revealed enrichment of miR-30c-5p validated targets related to different cancer pathways such as p53 and Wnt signaling pathways, etc. Finally, 31 gene targets, negatively correlated to miR-30c-5p tissue expression (TCGA data), were shown to possess clinical significance as their high expression determined shorter overall survival (OS), when considered as a group. All of these findings suggest that miR-30c-5p has the potential to serve as a novel, non-invasive biomarker for OSCC detection.

## 2. Materials and Methods

### 2.1. Patient Cohort and Saliva Collection

Saliva samples were obtained from 33 patients with histologically proven OSCC at the Department of Maxillofacial Surgery, University Hospital St. George, Plovdiv, Bulgaria (UHSG cohort). Both tumor tissue and non-tumorous epithelium at least 2 cm away from the tumor were collected from each patient as well. None of the patients had radiotherapy or chemotherapy or both before saliva collection. Clinical parameters including age, sex, social history, pathological features, TNM stage, therapy, and overall survival were prospectively collected (Table 1). The tumor stage was determined according to the 2002 Union for International Cancer Control TNM staging system. In addition, 12 healthy volunteers, with absence of any clinical signs for oral lesions and matched for age, gender, and risk factors were selected as normal controls. The study was approved by the Institutional Ethics Committee of Medical University-Plovdiv (Protocol №1/25.02.2016), and all patients provided written informed consent.

OSCC patients and healthy volunteers were requested to avoid smoking, eating, drinking, and oral hygiene procedures for at least 1 h prior to saliva collection. Each participant was asked to rinse their mouth with distilled water 2 times and then wait for 5 min prior to saliva collection (usually between 8 and 9 a.m.). Unstimulated whole saliva (around 4–5 mL) was collected after spitting into chilled 50 mL tubes for no more than 30 min. Immediately after, the saliva was processed by centrifugation at 2600× *g* for 15 min at 4 °C to remove cellular components, bacteria, and any food residuals. The cell-free RNA containing supernatant was aliquoted (500 µL) and stored at −80 °C until further RNA extraction. As a validation set, the data of OSCC patients at Medical Oncology, ASST Spedali Civili di Brescia, Brescia, Italy (SCUB cohort), previously described by Romani and colleagues [24], were kindly provided to the authors. The dataset included microarray analysis performed on 50 OSCC saliva samples and 42 samples from healthy subjects.

### 2.2. Total RNA Extraction and miRNA TaqMan Assay

Salivary cell-free RNA was extracted by using TRI Reagent (Invitrogen, ThermoFisher Scientific, Massachusetts, MA, USA) according to the manufacturer’s instructions. The concentration of RNA was determined spectrophotometrically on NanoDrop2000 (Thermo Scientific, Massachusetts, MA, USA). To analyze the expression of miR-21-5p, miR-34-3p, miR-193-5p, miR-376a-3p, miR-24-3p, miR-30c-5p, miR-125a-3p, and miR-214-3p, 30 ng RNA was retro-transcribed with the TaqMan microRNA Reverse Transcription Kit (Thermo Fisher Scientific, Massachusetts, MA, USA) and specific stem-loop 5x primers for each miRNA. The reverse transcription program included the following steps: 16 °C (30 min); 42 °C (30 min); 85 °C (5 min); 4 °C (~). Real time-PCR of miRNA expression was carried out in a final volume of 10.5 μL with TaqMan Advanced master mix (5.03 µL) (Thermo Fisher Scientific, Massachusetts, MA, USA), TaqMan MicroRNA^®^ Assays 20x primers (0.47 µL) (Thermo Fisher Scientific, Massachusetts, MA, USA), and 1:7 diluted cDNA (5 µL). Each sample was run in duplicates for analysis on a Rotor-Gene Q real-time PCR detection system (Qiagen, Hilden, Germany). The amplification protocol included an initial activation step at 50 °C (2 min), followed by denaturation at 95 °C (20 s) and 45 cycles at 95 °C (1 s) and 60 °C (20 s). The data were normalized to RNU6 as endogenous control. Relative miRNA abundance was calculated by the comparative 2^−ΔΔCt^ method and normalized to the RNU6 gene levels. R with ggplot2 and Cytoscape was used to present the data.

### 2.3. HPV Detection in Tumor Specimens

For analysis of HPV status of the UHSG cohort, DNA was extracted from tumor tissue samples with TRI Reagent (Sigma- Aldrich, Missouri, MI, USA) according to the manufacturer’s instructions. The exact DNA concentrations were determined spectrophotometrically on NanoDrop2000 (Thermo Scientific Massachusetts, MA, USA). The absence of 19 medium-high risk HPV strains (HPV16, -18, -26, -31, -33, -35, -39, -45, -51, -52, -53, -56, -58, -59, -66, -68, -69, -73, and -82) was confirmed by the strip hybridization test (Operon, Zaragoza, Spain). DNA quality was established by amplification of a fragment of the human β-globin gene localized on the strip.

### 2.4. Discrimination Power Analysis

The biomarker properties of miR-30c-5p for OSCC diagnosis were evaluated by the receiver operating characteristic (ROC) curve. ROC was designed on the basis of the expression levels of miR-30c-5p in controls and OSCC patients from the SCUB cohort by MATLAB R2020b. All ROC-related parameters, including sensitivity, specificity, area under curve (AUC), and *p* value were calculated by the software. Youden’s index (sensitivity + specificity-1) was used to select the optimal threshold value indicated as a red square.

### 2.5. TCGA Dataset of OSCC

We used OSCC patient cohorts of the Broad Institute TCGA Genome Data Analysis Center (http://gdac.broadinstitute.org/ (accessed on 28 January 2016)): Firehose stddata__2016_01_28. Broad Institute of MIT and Harvard (OSCC N = 354 and Normal N = 44) to evaluate miR-30c-5p in both normal and oral cancer tissue samples. Significance of miR-30c-5p and gene modulation between expression values of normal and tumor samples was assessed by Wilcoxon test. Significance was defined at the *p* < 0.05 level.

### 2.6. miR-30c-5p Target Prediction and Pathway Enrichment Analysis

The MiRNet (https://www.mirnet.ca/miRNet/home.xhtml (accessed on 1 May 2021)) web tool, based on Tarbase v8, was used to determinate miRNA-target interaction and pathway enrichment analysis. A Pearson’s correlation coefficient was used to establish significance of negative association on patient samples for each validated miR-30c-5p-target interaction. The analyses were conducted entirely with Matlab R2020b (The MathWorks Inc., Massachusetts, MA, USA, http://www.mathworks.com).

### 2.7. Survival Analyses

Overall survival (OS) were estimated by using Kaplan–Meier analysis, and the log-rank test was used to assess differences between curves. A multivariate Cox proportional-hazards regression model was built to evaluate the effect of clinical variables on survival analysis. Patients with high and low signal intensity were defined by considering positive and negative z-score values, if not differently specified. The analyses were conducted entirely with Matlab R2020b.

## 3. Results

### 3.1. miR-30c-5p Is Weakly Expressed in OSCC Saliva

**Figure 1:**
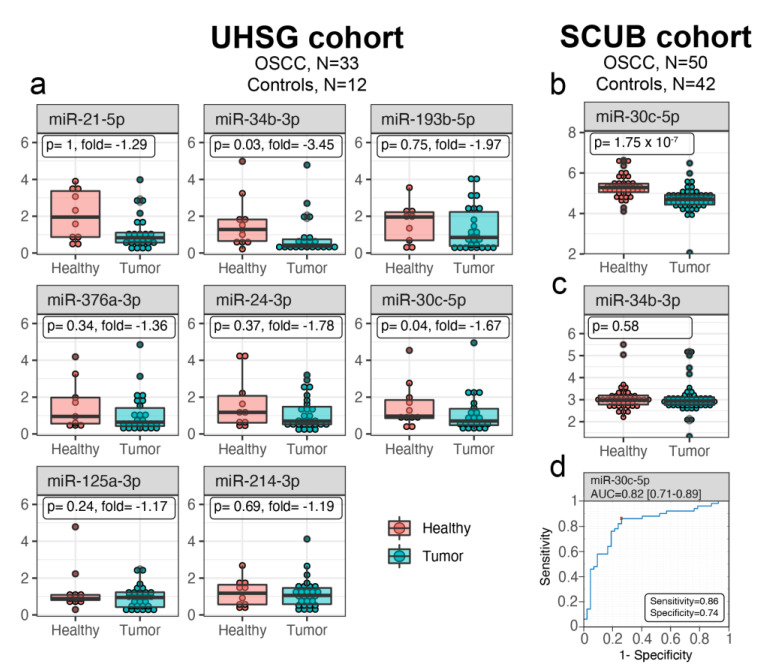
miR-30c-5p is depleted in OSCC saliva and can be used for differentiation of cancerous from normal patients. (**a**) Box plots of miR-21-5p, miR-34-3p, miR-193-5p, miR-376a-3p, miR-24-3p, miR-30c-5p, miR-125a-3p, and miR-214-3p expression, evaluated in both OSCC and normal saliva from the UHSG cohort. Expression levels were determined by TaqMan assay after total RNA extraction from saliva and presented as fold difference. Data are summarized from two technical replicates for each patient. Box plots showing expression levels of miR-30c-5p (**b**) and miR-34-3p (**c**) in normal control and OSCC saliva from the SCUB cohort. The expression was measured by miRNA microarray assay on RNA obtained from each sample of tumoral and normal saliva and presented as fold difference. (**d**) Diagnostic performance of miR-30c-5p for OSCC. ROC curve analysis of salivary miR-30c-5p in discriminating between patients and healthy individuals from the SCUB cohort. The red square indicates the optimal cutoff threshold value for diagnosis.

miRNAs are highly abundant in saliva, and their transcriptional changes might serve as a warning message for development of different malignancies, including OSCC. In order to identify OSCC-specific salivary miRNAs, the transcription levels of a panel of 8 cancer-related miRNAs (miR-21-5p, miR-24-3p, miR-30c-5p, miR-34-3p, miR-125a-3p, miR-193-5p, miR-214-3p, and miR-376-3p) was measured in 33 OSCC HPV-negative and 12 control preoperative salivary samples from the UHSG cohort (Bulgaria) by TaqMan assay. The miRNAs were selected on the basis of a literature review, underlining their role in different cancers, including OSCC. The expression of these miRNAs is presented as boxplots in Figure 1a. Among the analyzed miRNAs, the transcriptional levels of miR-30c-5p (*p* = 0.04) and miR-34-3p (*p* = 0.03) were found to be significantly downregulated only in OSCC saliva. To validate the primary results regarding the transcriptional behavior of miR-30c-5p and miR-34-3p, the expression of both miRNAs in saliva samples from patients enrolled in the SCUB cohort (Italy) was checked. The dataset included global miRNA microarray analysis performed on salivary samples obtained from 50 OSCC and 42 healthy subjects [24]. Our validation study showed that only miR-30c-5p (*p* < 0.001) was underexpressed in the SCUB cohort OSCC saliva (Figure 1b). These data confirmed the results of the UHSG cohort, whereas miR-34-3p (*p* < 0.58) showed no changes (Figure 1c).

Usually, saliva is in direct contact with the oral cancer tissues. Moreover, the tumor cells secrete a variety of molecules, including miRNAs, directly into the saliva. Because the expression of miR-30c-5p was down-regulated in saliva from OSCC patients from two different cohorts (83 OSCC and 54 controls in total), the expression levels of the miR-30c-5p were further evaluated in tumor compared to non-tumor tissues in the TCGA data set (354 OSCC and 44 controls). Contrary to the results from saliva, the relative expression of miR-30c-5p was significantly (*p* < 0.001) higher in TCGA OSCC tissue samples than in their matched non-tumor tissues (data not shown). It could be assumed that OSCC tissues were not the source for miR-30c-5p in saliva.

Altogether, the results from two different patient cohorts revealed that miR-30c-5p was downregulated in saliva of patients with OSCC compared to healthy subjects. However, it was most probably not secreted directly by the tumors.

### 3.2. Salivary miR-30c-5p Has the Potential to Discriminate OSCC Patients from Healthy Controls

To determine whether the downregulation of miR-30c-5p can distinguish patients with OSCC from healthy controls, an ROC curve was constructed to evaluate the discrimination power of this miRNAs as a potential biomarker for OSCC diagnosis. The designed ROC curve for miR-30c-5p expressed the sensitivity (true positive rate) versus 1-specificity (false positive rate) at various cut-off values and the AUC, indicating the discrimination power of this miRNA (Figure 1d). The red square on the figure represents the Youden’s index (sensitivity + specificity-1) which is the optimal cutoff threshold value for diagnosis. Comparing OSCC subjects with controls, AUC of miR-30c-5p was found to be 0.82 (95% CI: 0.71–0.89). The sensitivity and the specificity of miR-30c-5p were 86% and 74%, respectively. Taken together, these findings demonstrate that salivary miR-30c-5p levels have great potential as a diagnostic biomarker in OSCC.

### 3.3. miR-30c-5p Target Genes Are Involved in p53 and Wnt Signaling Pathways in OSCC

**Figure 2:**
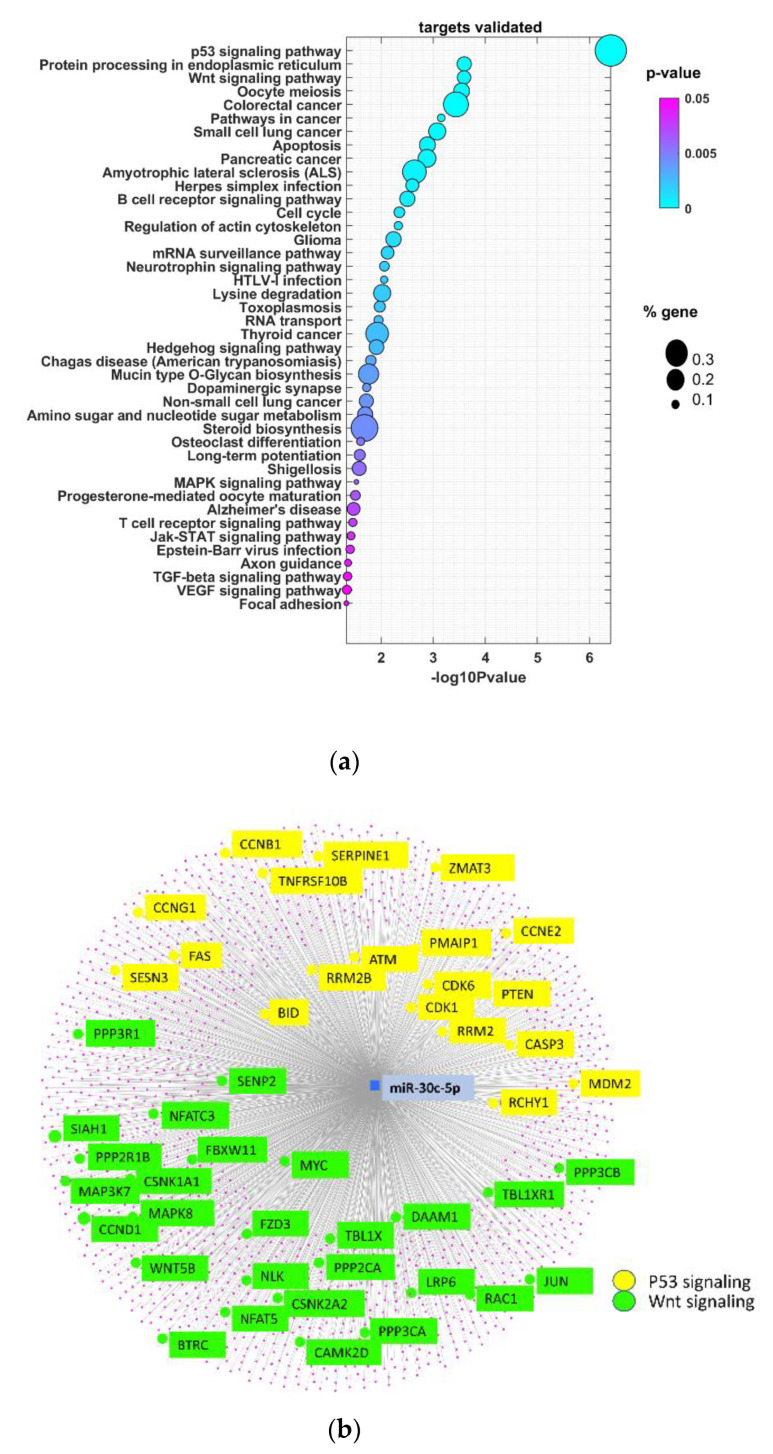

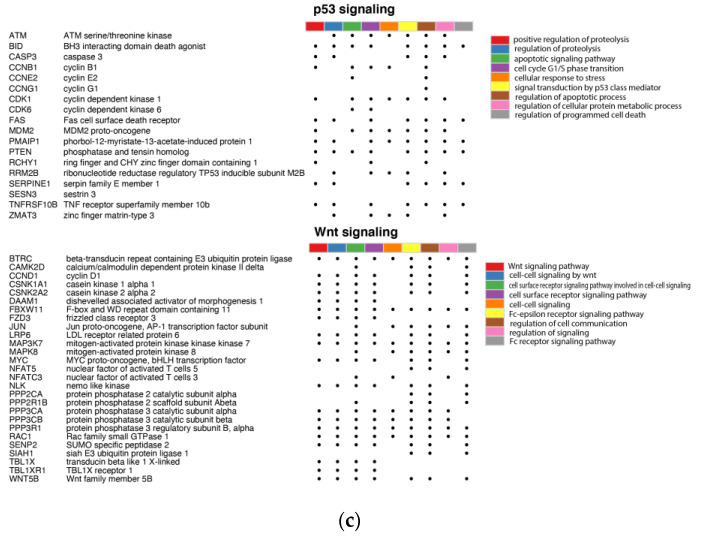
miR-30c-5p target genes are abundant in p53 and Wnt signaling pathways in OSCC. (**a**) Bubble plot containing specific ontological groups of miR-30c-5p validated targets. The graphs show only the GO groups above the established cut-off criteria (*p* with correction <0.05, minimal number of genes per group >10). The size of each bubble reflects the number of differentially expressed genes assigned to the GO terms. The transparency of the bubbles displays the *p*-values (more violet is closer to the border of *p* = 0.05). (**b**) Graphical view of a network built by the validated miR-30c-5p targets. Targets that belong to p53 and Wnt signaling pathways are highlighted. p53-related genes are marked in yellow, and Wnt are marked in green. (**c**) Detailed overview of target genes involved in regulation of p53 and Wnt signaling pathways. Symbols and gene names are presented with the nine ontological groups with the lowest *p*-value from the GO BP (gene ontology biological process) database. Individual GO BP categories are marked with an appropriate color. A dot indicates the assignment of a given gene to a specific category.

miRNAs exert their regulatory role mainly by binding to their target mRNA, causing its degradation or translational inhibition. Accordingly, the miRNet web tool was used to identify validated protein-coding genes controlled by miR-30c-5p. We identified 1767 validated targets (Supplementary Table S1). Next, the biological processes regulated by miR-30c-5p targets were determined (Figure 2a). As a result of this functional enrichment analysis, significant enrichment in 42 GO processes (*p* < 0.05) was demonstrated. Among them, different pathways in cancer, including the p53 and Wnt signaling pathways, were found to be most commonly enriched. p53 represents the most frequently mutated gene in HNSCC, with a frequency of 72% [25]. Moreover, miR-30c has been demonstrated to directly bind p53 3′UTR, inhibiting its translation. miR-30c and miR-181a synergistically modulate the p53–p21 pathway in diabetes-induced cardiac hypertrophy. Although there is no direct evidence of genetic alteration in the Wnt pathway in head and neck tumors, several works demonstrated that this pathway might be over-induced as a results of trans activation of EGFR and PI3K signaling [26]. In that context, the inhibitory effect of miR-30c-5p on the p53 and Wnt pathways could represents an important event during the onset and development of OSCC. Figure 2b represents a graphical view that includes the number of miR-30c-5p validated targets participating in p53 and Wnt signaling pathways in OSCC. The detailed analysis of miR-30c-5p validated target genes from each pathway showed their role in the regulation of different biological processes, with most representative, inter alia: for p53-, “regulation of proteolysis”, “apoptotic signaling pathway”, “cellular response to stress”, “regulation of cellular protein metabolic process”, and for Wnt, “cell surface receptor signaling pathway”, “cell-cell signaling”, “regulation of cell communication”, “Fc receptor signaling pathway” (Figure 2c). Taken together, these results indicate that miR-30c-5p target genes are involved in p53 and Wnt signaling pathways in OSCC.

### 3.4. miR-30c-5p Targets Have Prognostic Value in OSCC

**Figure 3:**
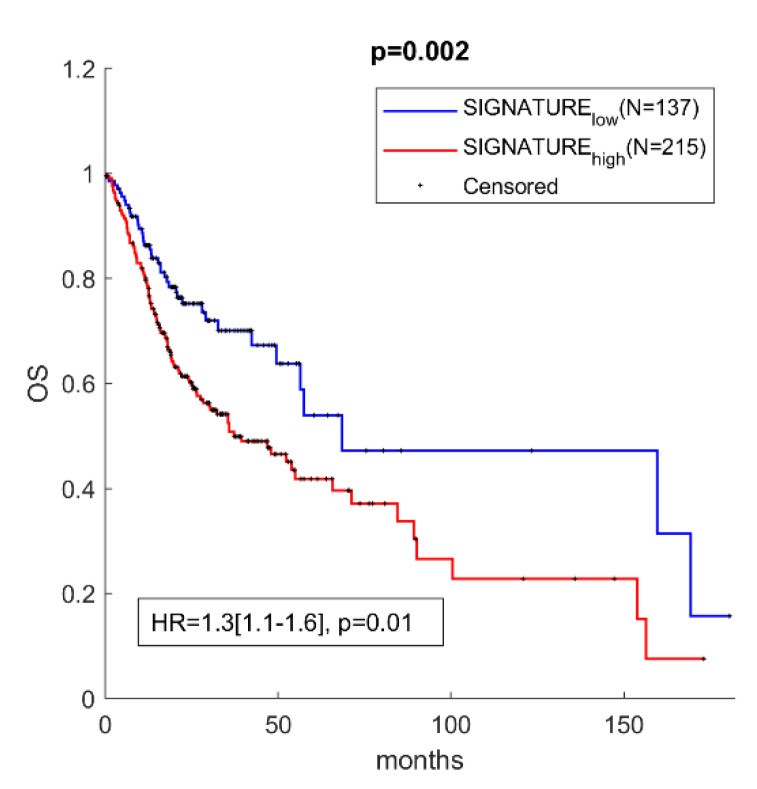
High expression of miR-30c-5p targets had prognostic value in patients with OSCC. Kaplan–Meier analysis representing the correlation between expression levels of miR-30c-5p targets considered as a group and OS in OSCC patients from the TCGA cohort. The TCGA cohort was divided in two subgroups according to positive and negative z-scores of the mean expression level of each prognostic mRNA. For each KM curve, the hazard risk, confidence interval, and relative *p*-value (*p*) of the multivariate Cox analysis are also indicated.

Next, a decision was made to investigate if miR-30c-5p targets might have prognostic value and can be used as a predictive indicator for OS in OSCC. In order to test this, a score was assigned to each patient from OSCC TCGA. Then, based on the expression levels of miR-30c-5p targets, a Kaplan–Meier analysis was carried out comparing patients with positive and negative scores. Among the 1767 validated targets, those with significant negative correlation (Pearson’s R) to the miR-30c-5p and with individual prognostic value were chosen. The final list of 31 selected targets can be found in Supplementary Table S2. The overall signature of the 31 targets was then used to assign a score to each patient from OSCC TCGA. Then, based on the expression levels of miR-30c-5p targets, a Kaplan–Meier analysis was carried out comparing patients with positive and negative scores. As presented in Figure 3, the survival analysis showed that high expression of miR-30c-5p targets (gene signature high) was predictive of shorter OS in the TCGA cohort, compared to patients with negative scores (gene signature low). These results highlighted the prognostic power of miR-30c-5p targets when they were used as a signature in OSCC patients.

## 4. Discussion

Irrespectively of the implementation of several screening and awareness programs, OSCC incidence still remains high. The tumor stage at the time of diagnosis is the main factor that determines the 5-year survival rate in OSCC. Accordingly, early detection has a much more favorable outcome and leads to better survival of the patients in comparison to those diagnosed at advanced stages. Examination of the suspected lesions followed by tissue biopsy and repetitive blood sampling are the routine procedures used for OSCC diagnosis. However, they have several drawbacks as they are time-consuming, physically intrusive, and cause excessive stress and pain, which altogether lead to poor patient compliance. Moreover, the tumor heterogeneity is another aspect that cannot be addressed by the above-mentioned procedures, as patients with tumors of the same clinical-pathological stage do not necessarily have the same disease progression, response to treatment, rate of disease recurrence, and survival. As surgery is typically the first-line treatment for early diagnosed oral cavity tumors, patients with cancers treated in their early stages may have little posttreatment disfigurement. For patients diagnosed at a later stage, surgical removal may necessitate facial reconstruction. Adjunctive therapy may also be required to assist in speech, chewing, and swallowing; fabrication of dental or facial prostheses may also be needed [27,28]. Recently, salivary diagnostics is becoming more popular among clinicians and scientists as it is noninvasive, easily performed, and cost-effective. Indeed, the power of saliva as a medium for detection of DNA, RNA, proteins, and metabolites as biomarkers of different malignant diseases has been shown [29]. Therefore, the identification of new salivary biomarkers has the potential to improve OSCC diagnosis, predict the development of relapses and the response to therapy, or monitor the patient’s outcome.

Detection of miRNAs in saliva as biomarkers for different diseases [29,30] and more specifically, for oral cancer [31], has been published by few groups. The present study was driven by the fact that not many OSCC-specific, salivary miRNAs have been reported in the literature. In this study miR-30c-5p was identified in human preoperative OSCC saliva, and its diagnostic properties were further explored. First, the transcription levels of a panel of 8 cancer-related miRNAs (miR-21-5p, miR-34-3p, miR-193-5p, miR-376a-3p, miR-24-3p, miR-30c-5p, miR-125a-3p, and miR-214-3p) were measured in 33 OSCC HPV-negative preoperative and 12 control salivary samples from a UHSG cohort (Bulgaria), and among them, a significant downregulation of miR-30c-5p and miR-34-3p in tumor saliva was found. Further validation in another 50 OSCC and 42 healthy subjects (SCUB cohort, Italy) revealed that only miR-30c-5p showed the same transcriptional behavior, whereas miR-34-3p had no changes. For this reason, only miR-30c-5p was selected for further studies.

The advantage of screening miRNAs in OSCC preoperative saliva, aiming for an early diagnosis, is further supported by the fact that this biological fluid is in direct contact with the cancer tissues in the mouth. As we found that miR-30c-5p was underexpressed in saliva from OSCC patients from two different cohorts (83 OSCC and 54 controls in total), we hypothesized that the tumor tissues by themselves could be the source of this miRNA. However, the TCGA analysis (354 OSCC and 44 controls) showed that miR-30c-5p was significantly (*p* < 0.001) higher in OSCC tissue samples than in their matched non-tumor tissues. This observation is in contrast to the results obtained from salivary samples. It suggests that miR-30c-5p is most probably not secreted directly by the tumor cells. However, this conclusion is rather uncertain as we have not performed postoperative miR-30c-5p analysis.

To better evaluate the discrimination power of downregulated miR-30c-5p in OSCC patients from healthy subjects, AUC was found to be 0.82 (95% CI: 0.71–0.89), with sensitivity and specificity of 86% and 74%, respectively. Moreover, as we analyzed saliva from oral cancer patients without any other malignancies, we could speculate that miR-30c-5p expression is OSCC-specific. These findings demonstrate that salivary miR-30c-5p could be suggested as a new biomarker for OSCC, which can be validated through further studies on different and larger patient cohorts. However, the possible role of miR-30c-5p as a biomarker for early diagnosis or development of recurrence needs further investigation.

miR-30c-5p studied here has been shown to be involved in other cancers such as colorectal cancer [32,33], non-small cell lung cancer [34,35], gastric cancer [36], pancreatic ductal adenocarcinoma [37], renal cell carcinoma [38,39], hepatocellular carcinoma [40,41,42,43], breast cancer [44,45,46,47,48], laryngeal cancer [49], and other malignancies. For example, in gastric cancer, miR-30c-5p suppresses migration, invasion, and epithelial to mesenchymal transition via targeting *MTA1* [36]. It plays an onco-suppressive role in clear cell renal cell carcinoma by targeting *HSPA5*, which leads to inhibition of the progression of the disease, both *in vitro* and *in vivo*. Additionally, in laryngeal cancer, *DLEU2* enhances the malignant properties of the cancer cells via the miR-30c-5p/*PIK3CD*/*Akt* axis [49].

The pathway enrichment analysis based on validated miR-30c-5p targets showed significant enrichment of these genes in 42 GO processes. Specifically, a number of those targets were shown to participate in essential oral cancer signaling pathways—p53 and Wnt. p53 genetic mutations are a common occurrence in HNSCC. We have previously identified several miRNAs associated with both p53 mutation status and overall and disease-specific survival in oral cancer patients [50]. However, HNSCC tissue samples were used, but not saliva. It seems promising to be able to identify a miRNA in saliva that is involved in the p53 signaling pathway, so common in oral cancer. It is well known that Wnt signaling/β-catenin pathway dysregulation is involved in cancer genesis, including oral cancer. This pathway is accompanied by various mechanisms, such as epithelial-mesenchymal transition and involvement of various miRNAs [51,52]. Participation of miR-30c-5p targets in this signaling pathway as shown by the pathway enrichment analysis underlies its role in oral cancer development. Accordingly, its expression in tumor tissue, as well as its reduction in saliva specimens, could represent an early event of oral carcinogenesis, thus highlighting its relevance as a diagnostic biomarker.

Also, the miR-30c-5p targets have clinical significance, as high expression levels of the gene signature (positive z-score) are predictive of shorter OS in TCGA cohorts compared to patients with a negative score.

Some of the miR-30c-5p targets have been shown to regulate OSCC growth, metastasis, and radioresistance. For example, Serpin family E member 1 (*SERPINE1*) is a serine proteinase inhibitor (serpin) that frequently has cancer-specific induction. Zhao et al., demonstrated that *SERPINE1* is overexpressed in both OSCC tissues and cell lines. *SERPINE1* silencing is followed by impaired OSCC cell viability and proliferation as a result of apoptosis [53]. Another miR-30c-5p target, Yin Yang 1 (*YY1*), is also overexpressed in oral cancer patient samples compared to adjacent normal tissue. Both in vitro and in vivo experiments revealed pro-proliferative, pro-angiogenic, and pro-metastatic roles of *YY1* in oral cancer [54]. In addition, higher expression of *YY1* was observed in radioresistant cells and tissues. These results were further confirmed by *YY1* knockdown, which sensitized tongue cancer cells to ionizing radiation. The exact mechanism showed that the overexpression of *YY1* leads to upregulation of nuclear *PTEN* and *Rad51* expression, followed by more efficient DNA repair [55]. HEAT repeat-containing protein 1 (*HEATR1*) interacts with Pontin/Reptin, which leads to mTOR activation and pre-rRNA synthesis, finally resulting in enhanced OSCC cell proliferation. Expression studies confirmed high *HEATR1* levels in tumor tissues [56].

The use of miR-30c-5p as a non-invasive OSCC biomarker may be of relevance for (i) identifying a precancerous state at high risk of evolving to cancer even in patients with no clinically detectable oral potentially malignant disease; (ii) detection of OSCC at early stages; (iii) early detection of local recurrence or minimal residual disease; and (iv) the planning of scheduled follow-up visits and treatment strategy choices for OSCC patients. Moreover, liquid biopsy analysis has emerged as a minimally invasive approach to characterize the evolution of a solid tumor in real time. As miRNAs remain stable in bodily fluids and may reflect the characteristics of tumor and/or premalignant lesions, miR-30c-5p may be an excellent non-invasive biomarker for assessing cancer progression, especially for cases in which tissue is not available. Further on, diagnostic tests based on liquid biopsies could be applied in screening programs for high-risk individuals such as heavy smokers, severe alcohol consumers, and HPV-positive patients. In summary, the proposed approach has the potential to significantly improve patients’ long-term survival, reduce morbidity, and minimize the extent of the surgical treatment required.

## 5. Conclusions

In the present work, we demonstrated the use of preoperatively detected salivary miR-30c-5p as a non-invasive way to differentiate OSCC patients from healthy individuals. The proposed approach promises to be more reliable and sensitive in comparison to visual and tactile screening and to potentially revolutionize the diagnosis and monitoring of OSCC. Although miR-30c-5p shows its potential to serve as an OSCC-specific biomarker, further research on larger patient cohorts is needed to validate its application in clinical practice. Moreover, future investigation of the functional role of miR-30c-5p in oral cancer cell lines will shed new light regarding the mechanisms through which this miRNA performs its oncogenic role in this type of malignancy. Taken together, our results suggest potential biomarker properties for miR-30c-5p in OSCC.

## Figures and Tables

**Table 1:** Characteristics of OSCC UHSG cohort and controls included in the study.

Characteristics	OSCC Patients	Controls
Number	33	12
Gender	M 27 (81.8%)/F 6 (18.2%)	M 7 (58.3%)/F 5 (41.7%)
Age	61.61	54.17
Range	47–80	40–69
Smokers	29 (87.9%)	10 (83.3%)
HPV positivity	No	N/A
Histology	SSC	N/A
Location	Tongue 7 (21%)	
	Floor of mouth 15 (45.5%)	N/A
	Retromolar triangle 1 (3%)	
	Gingiva 10 (30.3%)	
Size (mm)	36,1	N/A
Range (mm)	10–70	

The table includes the total number of enrolled subjects, number and percentage of males and females, the median age of each group, the minimal and maximal age of the participants, tobacco use, HPV status, histology and tumor location, size and range only for the OSCC group. Abbreviations: M = male; F = female; HPV = human papilloma virus; SCC = squamous cell carcinoma.

## Data Availability

The data presented in this study are available on request from the corresponding author. The data are not publicly available due to privacy restrictions.

## Supplementary Materials

The following are available online at https://www.mdpi.com/article/10.3390/biomedicines9091079/s1, Table S1: List of 1767 validated miR-30c-5p target genes. Table S2: List of 31 validated miR-30c-5p target genes with relevance to OS.
